# Capric Acid-Based Therapeutic Deep Eutectic Systems: A Focused Review Within the Framework of Deep Eutectic Solvents

**DOI:** 10.3390/ph19010159

**Published:** 2026-01-15

**Authors:** Faisal Al-Akayleh, Ahmed S. A. Ali Agha, Ali R. Olaimat, Giuseppe Biagini

**Affiliations:** 1 Faculty of Pharmacy and Medical Sciences, University of Petra, Amman 11196, Jordan; 2 Department of Pharmaceutical Sciences, School of Pharmacy, The University of Jordan, Amman 11942, Jordan; ahm9220505@ju.edu.jo; 3 Health Innovative Products and Technologies (HIP-TECH) PhD Program, Department of Life Sciences, University of Modena and Reggio Emilia, 41125 Modena, Italy; 4 Department of Biomedical, Metabolic and Neural Sciences, University of Modena and Reggio Emilia, 41125 Modena, Italy

**Keywords:** therapeutic deep eutectic systems (THEDES), CA, deep eutectic solvents (DES), fatty acid-based eutectic systems, hydrogen bond interactions, artificial intelligence

## Abstract

**Background/Objectives**: Capric acid (CA)–therapeutic deep eutectic systems (THEDES) are emerging as a distinct class of biofunctional matrices capable of reshaping drug solubilization, permeability, and bioactivity. **Methods**: Relevant studies on CA–THEDES were identified through targeted database searches and screened for evidence on their design, mechanisms, and pharmaceutical performance. **Results**: This review synthesizes current evidence on their structural design, mechanistic behavior, and pharmaceutical performance, revealing several unifying principles. Across multiple drug classes, CA consistently drives strong, directional hydrogen bonding and drug amorphization, resulting in marked solubility enhancement and stabilization of non-crystalline or supersaturated states relative to crystalline drugs or conventional solvent systems. Its amphiphilic C10 chain further contributes to membrane fluidization, which explains the improved transdermal and transmucosal permeation repeatedly observed in CA-THEDES. Additionally, synergistic antimicrobial and anticancer effects reported in several systems confirm that CA acts not only as a solvent component but as a bioactive co-therapeutic. Collectively, the reviewed data show that CA serves as a structurally determinant element whose dual hydrogen-bonding and membrane-interacting roles underpin the high pharmaceutical performance of these systems. However, gaps remain in long-term stability, toxicological profiling, and regulatory classification. Emerging Artificial Intelligence (AI) and Machine Learning (ML)-guided predictive approaches offer promising solutions by enabling rational selection of eutectic partners, optimal ratios, and property optimization through computational screening. **Conclusions**: Overall, CA-THEDES represent a rationally designable platform for next-generation drug delivery, where solvent functionality and therapeutic activity converge within a single, green formulation system.

## 1. Introduction

The growing demand for environmentally responsible and pharmaceutically acceptable alternatives to conventional organic solvents has driven significant interest in the development of advanced solvent systems. Traditional organic solvents are widely used in pharmaceutical processes; however, their volatility, toxicity, and poor biodegradability pose risks to both human health and the environment [1]. These concerns have accelerated the investigation of alternative solvent systems that combine functional performance with improved safety and environmental profiles. Among the alternatives investigated, deep eutectic solvents (DESs) have emerged as attractive candidates due to their ease of preparation, low cost, and potential for biocompatibility [2,3]. In recent years, a subclass known as therapeutic deep eutectic systems (THEDESs) has gained prominence. These systems incorporate at least one component with inherent pharmacological activity, enabling them to function simultaneously as solvent and therapeutic agent [4]. This dual role offers distinct advantages in drug formulation, particularly for compounds with poor aqueous solubility, low permeability, or instability in conventional vehicles [5,6,7]. Among fatty acids explored in THEDES development, CA (decanoic acid, C10:0) is especially attractive because of its natural origin, safety, and ability to form eutectic mixtures with various active ingredients [8,9,10,11].

Despite increasing reports on fatty acid-based DESs, a comprehensive evaluation of CA-THEDESs remains lacking. The present review addresses this gap by providing a critical and focused analysis of CA-THEDESs, with emphasis on their design principles, preparation methods, physicochemical characteristics, and pharmaceutical relevance. Particular attention is given to their role in enhancing drug solubility and bioavailability, modulating release kinetics, and supporting novel drug delivery strategies. This review also discusses the underlying mechanisms of drug–solvent interactions, safety considerations, and regulatory challenges, with the aim of supporting further research and translation into clinical and industrial applications.

## 2. Background on DES and THEDES

The development of non-toxic, biodegradable, and functionally versatile solvent systems has become a central theme in green chemistry and pharmaceutical formulation. Among the solvent systems under active investigation, ionic liquids (ILs) and DESs have gained substantial attention. ILs consist entirely of organic cations and inorganic or organic anions and are typically liquid at or near room temperature [12]. While they exhibit attractive physicochemical properties—such as negligible vapor pressure, tunable polarity, and high solvation capacity—their limited biodegradability, cytotoxicity concerns, and relatively high cost have restricted their widespread application in biomedicine [13].

DESs, by contrast, are formed through the association of a hydrogen bond donor (HBD) and a hydrogen bond acceptor (HBA), resulting in an eutectic mixture with a melting point significantly lower than that of the individual components [8]. DESs can be readily prepared from a range of naturally derived components, including choline chloride, sugars, organic acids, and fatty acids, many of which are classified as Generally Recognized as Safe (GRAS) [14,15]. Due to their biocompatibility, low toxicity, and high solubilization potential, DESs are increasingly being explored in drug solubilization, extraction of bioactive compounds, biocatalysis, and pharmaceutical formulations [16,17,18].

An emerging extension of this field is the concept of deep eutectic systems (DESy). Unlike traditional DESs, which are prepared prior to application, DESy are formed in situ during the process of solubilization or formulation, with the target compound itself (e.g., an active pharmaceutical ingredient or bioactive molecule) actively participating in the eutectic interaction [19]. This innovation eliminates the need for pre-formed DESs, simplifying processing, reducing energy input, and enhancing formulation efficiency. DESy have already shown promise in areas such as prebiotic extraction and are increasingly being considered for pharmaceutical applications where solvent–drug synergy is desired [20].

**Figure 1:**
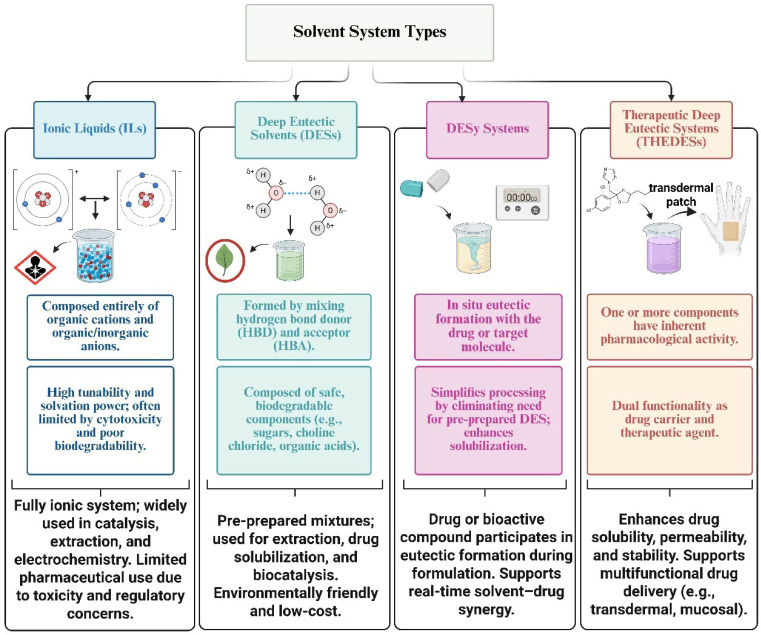
Comparison of ILs, DESs, DESy, and THEDESs based on composition, formation, and pharmaceutical function.

A particularly significant advancement within this family is the development of THEDESs, which are defined as eutectic systems in which one or more components possess intrinsic pharmacological activity [21]. THEDESs represent a novel class of multifunctional systems capable of acting simultaneously as drug carriers and therapeutic agents. Their dual functionality allows for improved drug solubility, controlled release profiles, enhanced membrane permeability, and the potential for synergistic therapeutic effects [5,6,7]. THEDESs also offer formulation advantages by reducing the need for conventional excipients and enabling novel routes of administration, including transdermal and mucosal delivery. As summarized in Figure 1, ILs, DESs, DESy, and THEDESs differ in their composition, method of formation, and pharmaceutical role, reflecting a clear progression from structurally defined solvents to multifunctional, therapeutically active systems.

To facilitate a clearer comparison of these solvent systems, Table 1 summarizes the fundamental differences between them, highlighting their respective compositions, interaction mechanisms, physicochemical properties, and pharmaceutical relevance.

## 3. CA as a Functional Component in Multicomponent Therapeutic Deep Eutectic Systems

**Figure 2:**
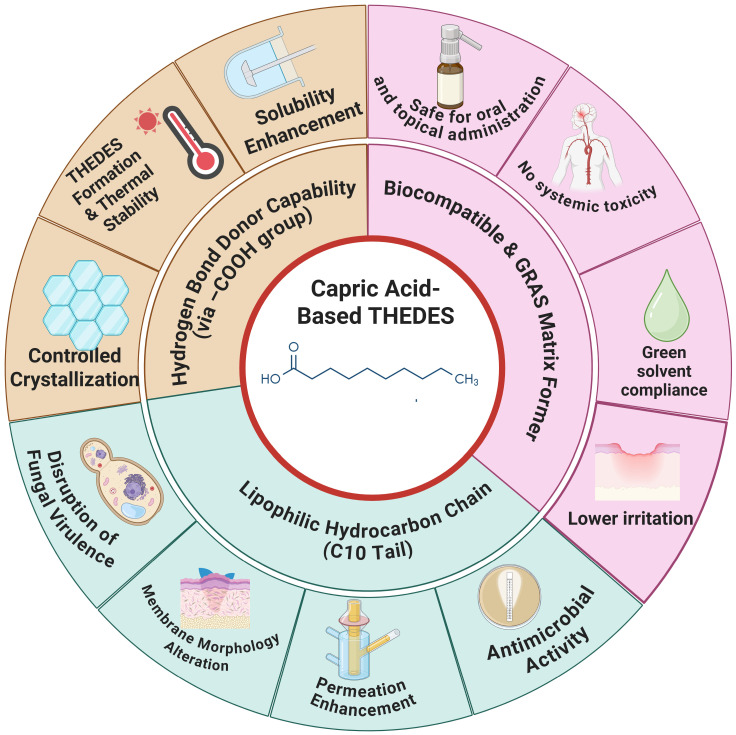
Schematic summary of the multifunctional roles of CA in THEDES.

CA is increasingly utilized as a HBD in the design of THEDES. As illustrated in Figure 2, its chemical structure—characterized by a terminal carboxylic acid and a saturated 10-carbon alkyl chain—enables core structural contributions that support its functional integration in THEDES: hydrogen bonding capacity [9], membrane-modulating hydrophobicity [25], and biocompatible matrix formation [26].

The carboxyl group forms extensive hydrogen bonding networks with various HBAs, including drugs and amino acids, thus facilitating eutectic formation, enhancing thermal stability, and significantly improving the solubility and amorphization of poorly water-soluble active pharmaceutical ingredients (APIs) such as ibuprofen, rasagiline, and lidocaine [7,8]. Additionally, its long alkyl chain imparts amphiphilic characteristics that enhance membrane interaction and lipid fluidization, which in turn promote transdermal and mucosal drug permeation and contribute to antimicrobial efficacy by disrupting microbial membranes [27,28]. These biophysical effects extend to antifungal applications, where CA inhibits *Candida albicans* virulence mechanisms, including hyphal formation and biofilm development [29], and induces lipid membrane remodeling such as tubulation [25]. From a formulation standpoint, CA’s GRAS classification and established biocompatibility make it an attractive choice for topical and oral routes, with in vivo studies confirming its low irritation and systemic safety in THEDES applications [8,30]. Notably, these properties are modulated by the physicochemical nature of the accompanying HBA, which plays a co-determinant role in defining viscosity, solubility, permeability, and biological performance of the final eutectic system. Therefore, CA’s performance as a THEDES component should be interpreted within the broader context of eutectic pair selection and structural complementarity—factors that are essential for tailoring systems to therapeutic and delivery-specific needs.

## 4. Preparation and Characterization of CA-THEDES

CA-THEDES are typically prepared through a simple, green synthesis route that relies on precise molar mixing of the HBD and HBA components under gentle heating and stirring [10,31]. Ratios are optimized based on phase diagrams to achieve eutectic formation, with systems becoming fully liquid at room temperature upon reaching the eutectic point [9].

Characterization of the eutectic nature and molecular interactions is routinely performed using thermal techniques such as Differential Scanning Calorimetry (DSC) and Thermogravimetric Analysis (TGA), revealing clear endothermic transitions and phase stability of the eutectic systems. In the Gefitinib/CA system, DSC confirmed a homogeneous liquid phase at the eutectic ratio, and TGA demonstrated thermal stability within acceptable pharmaceutical handling ranges [6]. FTIR spectroscopy consistently shows red-shifts in O–H and C=O vibrational bands, supporting extensive hydrogen bonding between carboxyl and hydroxyl groups of the HBD and HBA, respectively [9]. In addition, 1H and13C NMR confirm structural integration through chemical shift perturbations, particularly at the hydrogen bonding sites of APIs like Gefitinib and risperidone [6,31]. Phase behavior analyses indicate polymorphic transitions and suppressed crystallinity, a crucial factor in enhancing drug solubility. Morphological studies using microscopy further corroborate the homogeneity of THEDES formulations at room temperature [9]. Rheological assessments demonstrate that viscosity is highly tunable depending on molar ratio, temperature, and water content. Lower viscosity is generally favored to enhance drug diffusion and spreadability, and CA-based systems have shown viscosity reductions of up to 70% compared to conventional solvents [10]. Overall, CA-THEDES offers a highly tunable, biocompatible, and efficient platform for drug solubilization and delivery, provided that rigorous preparation and analytical characterization protocols are followed.

## 5. Role of CA in Promoting Real DESy Formation: Insights from Solid–Liquid Phase Diagrams

To date, there is no harmonized definition that clearly distinguishes DESy from ideal mixtures, despite this issue having been highlighted in the literature nearly five years ago [32]. In particular, existing definitions are contradictory: while thermodynamic criteria define a deep eutectic as a system whose experimental melting point is lower than the theoretical one—indicating a negative deviation from ideality—this interpretation is not applied consistently across the literature. Instead, many studies adopt a more functional definition, classifying systems as DES based on their practical performance, such as their ability to dissolve otherwise poorly soluble compounds, enhance permeability, improve stability, or act as green alternatives to conventional solvents supported by NMR, FTIR analysis [33,34].

A pivotal step in validating CA–THEDES is the comparison of theoretical and experimental solid–liquid phase diagrams. This analysis not only determines the eutectic composition but also reveals the extent to which CA drives systems toward genuine DES behavior rather than simple ideal eutectic mixing—a recurring concern among experts in the DES field. Across the investigated systems, CA consistently induces strong negative deviations from ideality, a defining thermodynamic signature of real DES formation and a performance level rarely achieved by many commonly used conformers.

In both the ketoconazole–CA (KCZ–CA) and clotrimazole–CA (CLOT–CA) systems, CA demonstrated a remarkable capacity to disrupt the ideal melting behavior predicted by theoretical models, leading to pronounced shifts in eutectic composition and substantial depressions in eutectic temperature. The KCZ–CA mixture exhibited a eutectic point at a KCZ molar fraction of 0.16, while the CLOT–CA mixture reached its eutectic composition at a CLOT molar fraction of 0.25. In each case, the experimental melting curves deviated significantly from ideal predictions, and the resulting mixtures readily transitioned into stable liquids at their eutectic ratios. These pronounced non-idealities reflect CA’s strong hydrogen-bond-donating capacity, favorable hydrophobic compatibility with lipophilic drugs, and its ability to substantially reduce drug crystallinity, resulting in physicochemical behavior that is distinct from and often more pronounced than that observed with longer-chain fatty acids or commonly used DES co-formers.

It is worth noting that similar pharmaceutical objectives have been pursued through co amorphous systems, where drug–coformer interactions stabilize amorphous solid dispersions. While co amorphous formulations enhance solubility and stability via solid state mechanisms, CA-THEDES achieve functional benefits through liquid state non ideality. This parallel underscores the importance of distinguishing between thermodynamic eutectic behavior and functional performance across different formulation strategies [35].

In conclusion, the observed negative deviations, melting-point depressions, and eutectic shifts across the drug–CA combinations suggest that CA may play a role in promoting non-ideal behavior in these systems. These trends indicate that CA could be a suitable coformer for the formation of DESs and may have potential as an excipient in the development of pharmaceutical THEDES with improved functional characteristics.

## 6. Pharmaceutical Applications

CA-THEDES have shown remarkable potential in addressing key formulation challenges, including poor drug solubility, low bioavailability, and suboptimal stability. By incorporating APIs into a eutectic matrix with CA, these systems can dramatically improve the dissolution of hydrophobic drugs.

For example, a eutectic of risperidone with CA achieved a 70,000-fold increase in solubility compared to water [27], while a gefitinib–CA THEDES (optimal 80:20 molar ratio) yielded a 30,000-fold solubility boost over aqueous media [6]. Such massive solubilization stems from strong hydrogen-bond interactions and amorphization of the drug within the fatty-acid matrix, effectively overcoming crystalline lattice energy.

This improved thermodynamic solubility translates into faster dissolution rates and can significantly enhance oral or topical bioavailability of poorly water-soluble compounds [36]. Furthermore, the microenvironment of the THEDES can stabilize labile drugs, as the hydrogen-bonded network and low water activity help prevent degradation pathways (e.g., hydrolysis or oxidation) [37]. The viscous, non-volatile nature of CA eutectics limits API exposure to oxygen, thereby reducing oxidative instability of sensitive molecules [37]. Overall, the capric-acid THEDES serves as a solvating and protective matrix that maintains drug molecules in a supersaturated yet stabilized state until administration.

Beyond solubility benefits, CA-THEDES have been shown to improve drug permeability and pharmacokinetic profiles. The amphiphilic CA moiety can fluidize biological membranes and act as a penetration enhancer, which is especially useful for transdermal and transmucosal delivery [37]. Likewise, risperidone-CA THEDES showed markedly higher skin permeation (with melting-point depression to ~17 °C at a 40:60 *w*/*w* ratio) and caused no irritation on rat skin [27]. In the case of droperidol (a sedative with low oral bioavailability), forming a THEDES with CA enabled a solvent-free liquid formulation that demonstrated superior intestinal absorption in ex vivo gut models [8]. The droperidol–CA system exhibited significantly higher flux across everted intestinal sacs compared to solid drug, highlighting how THEDES can enhance transmucosal delivery and potentially replace injectable administration with oral dosing.

Similarly, aripiprazole (an antipsychotic) was converted into a CA eutectic at certain ratios, yielding a clear, H-bond-stabilized liquid that avoids crystalline precipitation and is expected to improve oral uptake and onset of action [8]. These examples underscore that CA THEDES can tackle multiple biopharmaceutical hurdles simultaneously—increasing drug solubility, maintaining supersaturation, and facilitating membrane permeation—thereby boosting overall drug bioavailability.

Another important application of CA-based eutectics is in topical and transdermal drug delivery. Many active agents suffer from poor skin penetration or require high solvent content in creams. THEDES offers a way to combine the drug with a fatty acid that both solubilizes the API and enhances its diffusion through skin. A striking case is the CA–ketoconazole (KCZ) THEDES developed for fungal infections. Ketoconazole, an antifungal that is poorly water-soluble and limited in skin uptake, formed a room-temperature eutectic with CA (optimized at 1:5 molar ratio) that showed dramatic improvements in therapeutic performance [7]. The KCZ–CA eutectic exhibited a 6.2-fold higher transdermal flux in Franz cell studies compared to a commercial ketoconazole cream, and it halved the minimum inhibitory concentration (MIC) against *Candida albicans* (indicating enhanced antifungal potency).

Importantly, improved antimicrobial potency in THEDES should be interpreted using established interaction frameworks rather than inferred solely from reduced MIC in the eutectic. Mechanistically, the observed improvement can reflect (i) an additive effect, where CA contributes its own membrane-disruptive antimicrobial activity while the API retains its intrinsic activity, and/or (ii) an exposure-driven potentiation effect, where the eutectic increases apparent API activity by improving solubilization, partitioning, and delivery to the microbial membrane. In contrast, synergy requires quantitative demonstration that the combined effect exceeds the expected additive effect, commonly assessed for antimicrobials using checkerboard assays and the fractional inhibitory concentration index (FICI), where synergy is typically defined as FICI ≤ 0.5, additivity as FICI > 0.5–1.0, and indifference/antagonism at higher values depending on the interpretive scheme [38].

This dual enhancement—increased permeation and a 2× stronger antifungal effect—is attributed to the intimate hydrogen bonding between ketoconazole’s imidazole ring and CA’s carboxyl, which creates a stable liquid complex that both delivers more drug into the skin and leverages CA’s inherent antimicrobial activity.

In general, CA and other medium-chain fatty acids are known to disrupt microbial membranes; when used as part of a THEDES, they can synergize with antimicrobial drugs. For example, eutectic mixtures of capric with lauric or myristic acid show significant activity against Gram-positive bacteria (*S. aureus*, MRSA, *C. albicans*), including the ability to dissolve and remove biofilms by ~80–90% within minutes [39].

When combined with conventional antibiotics, such as in a CA–levofloxacin DES, the fatty acid can provide a complementary antibacterial effect and improve drug solubility, potentially overcoming resistance mechanisms [40]. This highlights the multifunctional role of CA in THEDES: it acts as a solvent, an absorption enhancer, and an active adjuvant (antimicrobial or anti-inflammatory) that can augment the pharmacodynamics of the co-formulated API.

Indeed, a gefitinib–CA THEDES not only increased gefitinib solubility (up to 30,000-fold in the reported system) but also reduced IC_50 values in EGFR-expressing cell lines relative to gefitinib and CA tested individually, indicating enhanced cytotoxic activity of the eutectic system [6]. Although the primary study describes this improvement as synergistic, the reported evidence is based on IC_50 comparisons at fixed compositions rather than formal interaction analysis; therefore, in this review the effect is interpreted conservatively as enhanced activity that may reflect additive capric-acid bioactivity and/or exposure-driven potentiation driven by markedly increased drug solubility and cellular accessibility, unless synergy quantification is explicitly reported

Such enhancement suggests that CA’s intrinsic bioactivity (e.g., mild anticancer effects and membrane-permeation facilitation) can contribute additively to the therapeutic outcome of the primary drug and/or potentiate its apparent efficacy through improved solubilization, cellular access, and local drug exposure.

CA-THEDES are also versatile in terms of dosage form design. Being liquids or low-melting semi-solids, they can be directly incorporated into various formulation types without traditional organic solvents [8]. Topical gels and ointments can be formulated by simply mixing a CA eutectic with gelling agents or emulsifiers, yielding drug-loaded gels that maintain high API solubility at skin temperature [4]. Transdermal patches and films have been developed by impregnating polymer matrices with drug–CA DES: for instance, incorporating a deep eutectic solution of a drug into an ethylcellulose film led to improved drug permeation and antifungal efficacy in a skin model [7]. Because THEDES can dissolve a large drug payload in a small volume, patch reservoirs or dissolvable films can deliver higher doses through the skin compared to conventional formulations.

Additionally, injectable depots represent an emerging application of DES technology. While CA-based systems have mostly been explored for oral and topical routes so far, the concept of using a biocompatible eutectic as a long-acting injectable is highly promising [8]. Deep eutectic formulations can be designed to be biodegradable, low-toxicity liquid implants that solidify or form microemulsions upon injection, thus slowing drug release [41]. A recent proof-of-concept in this arena employed a choline geranate-based deep eutectic mixture to create a subcutaneous depot for apomorphine, converting a thrice-daily injection therapy into an every-other-day regimen [42]. The depot, which self-emulsified in situ, entrapped the drug and extended its release over ~48 h. The inherent low volatility and mild melting point of CA eutectics make them stable under physiological conditions, and their Generally Recognized as Safe (GRAS) status suggests good biocompatibility [37]. Any residual fatty acid would be metabolized via normal lipid pathways. This approach could be transformative for depot delivery of drugs like antipsychotics or analgesics, reducing dosing frequency and improving patient compliance.

Collectively, CA-THEDES represent a novel and highly adaptable platform in pharmaceutics. They enable substantial and formulation-dependent solubility enhancement for poorly soluble drugs, stabilize active compounds within hydrophobic green solvent matrices, and in several cases improve drug release kinetics and transmembrane transport, as demonstrated by sustained liquid eutectic formation, enhanced intestinal flux, and improved transdermal permeation relative to non-eutectic or crystalline counterparts, as shown in Table 2.

## 7. Mechanisms of Drug–Solvent Interactions

**Figure 3:**
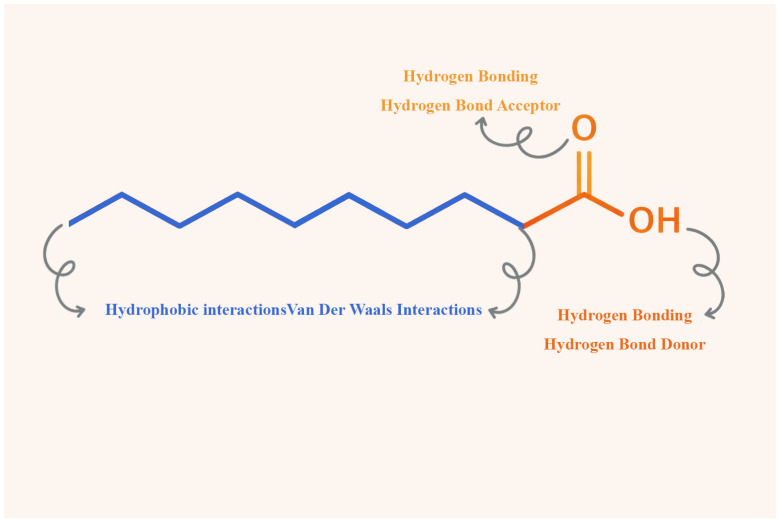
Molecular Interaction Sites of CA Involved in Hydrogen Bonding and Hydrophobic Interactions.

The molecular structure of CA, characterized by a terminal carboxylic acid group and a long hydrophobic alkyl chain, confers versatility in its interactions with both hydrophilic and hydrophobic moieties. As mentioned earlier, DESs are primarily formed through strong hydrogen bonding between a HBD and a HBA, which leads to lattice disruption and a marked decrease in the melting point of the resulting mixture. The carboxylic acid group in CA can function as both a hydrogen bond donor, through its hydroxyl hydrogen, and as a hydrogen bond acceptor, through carbonyl oxygen, facilitating extensive hydrogen-bonding networks within deep eutectic systems. The possible interaction sites of CA are illustrated in Figure 3.

Some of us also reported the successful fabrication of CA and menthol DES further enhancing the drug solubility of hydrophobic model drugs fluconazole and mometasone furoate. FTIR analysis suggested the contribution of CA carboxylic acid group as HBD and HBA Another notable structural change in the CA–menthol system was the disruption of the hydrocarbon side chains, indicating the presence of hydrophobic interactions, such as van der Waals forces [10,54].

Building on this work, Al-Akayleh et al. have fabricated CA–gefitinib DES, enhancing gefitinib aqueous solubility by 30,000-fold and confirmed CA hydrogen bond interactions through hydroxyl hydrogen and carbonyl oxygen as confirmed by FT-IR and NMR [6]. Furthermore, Alkhawaja et al. reported the fabrication CA aripiprazole and CA droperidol DESs reporting enhanced permeability, and NMR revelated CA interacts only via its –OH group of the carboxylic acid, acting exclusively as a hydrogen bond donor [8]. Understanding the nature of interactions between CA and other components of DES is vital, as it allows monitoring their impact on the characteristics of the final formulation.

At the molecular and biophysical level, the enhanced permeation observed with CA–based THEDES can be rationalized through a coupled partition–diffusion mechanism driven by membrane fluidization. The amphiphilic structure of CA enables preferential partitioning of the eutectic phase into lipid-rich domains of biological barriers, increasing local drug thermodynamic activity at the membrane interface [26]. Simultaneously, insertion of CA into lipid assemblies can disrupt acyl-chain packing, reduce lipid order, and increase free volume within the membrane, thereby lowering the energetic barrier for drug diffusion [55]. In the context of transdermal and transmucosal delivery, this translates into increased drug partitioning into the barrier followed by accelerated diffusion across a transiently disordered lipid matrix [26]. Importantly, these effects are formulation-dependent and arise from reversible physicochemical interactions rather than permanent membrane damage, consistent with the favorable tolerability reported for selected CA-based THEDES [6].

## 8. Comparative Evaluation

### 8.1. Relative to THEDES Based on Other Fatty Acids

There is limited literature comparing identical THEDES formulations of the same drug in which only the fatty acid component is varied, a strategy that enables investigation of the distinct effects of different fatty acids. To date, only one study has reported the fabrication of risperidone formulations using different ratios of CA, lauric acid, and myristic acid. That study demonstrated that both CA- and lauric acid-based systems successfully formed deep eutectic formulations, whereas myristic acid failed to do so. Among the successful systems, the CA-based THEDES exhibited a lower melting point than the lauric acid-based formulation, leading the authors to select the CA system for subsequent experimentation [27].

Fatty acids are commonly classified according to their degree of saturation or chain length. From a mechanistic standpoint, unsaturated fatty acids are often liquid at room temperature and may therefore behave more like solvents than true therapeutic deep eutectic system components. Additionally, the presence of cis double bonds can introduce structural kinks that may interfere with efficient molecular packing and intermolecular interactions [56,57]. However, these considerations are largely speculative and are not yet supported by systematic experimental comparisons in the literature. Accordingly, this section focuses on a theoretical discussion and comparison of the properties of saturated fatty acids relevant to deep eutectic system formation.

Short chain fatty acids are often referred to as volatile fatty acids due to their relatively low carbon number, low melting points, and higher vapor pressure, whereas longer chain saturated fatty acids have higher melting points and exist as solids at ambient conditions because of stronger van der Waals packing in a crystalline lattice. These physical property trends influence molecular mobility and therefore the ability to form stable deep eutectic systems [58,59,60].

The formation of deep eutectic systems is governed by intermolecular interactions and thermodynamic compatibility, with parameters such as carbon chain length, melting point, viscosity, and hydrophobicity playing key roles in determining the ability of components to form stable eutectic mixtures. Fatty acids can serve as a fixed component in such systems, either alone or in combination with other excipients, to propose formulations that facilitate the solubilization, permeation, and stabilization of the target drug [61].

The above-mentioned characteristics of medium-chain fatty acids are summarized in Table 3. As shown, CA presents a favorable balance between melting point, relevant viscosity, and hydrophobicity when compared to other medium-chain fatty acids [60,62,63].

Although these observations are theoretical, they suggest that CA may be particularly well suited for THEDES. Experimental validation is still required to confirm whether these properties explain the higher number and stability of capric-acid-based TDES reported in the literature.

### 8.2. Relative to Other Formulation Strategies

CA-THEDES offer notable advantages such as high drug loading potential, reduced excipient burden, and the possibility of synergistic therapeutic effects. However, they are highly sensitive to exact molar ratios, prone to phase instability and recrystallization, and suffer from limited toxicological data. Their hygroscopic nature complicates handling and scale up, and importantly, there are currently no marketed pharmaceutical products based on CA THEDES, with development still at the research stage [15,64].

Lipid based formulations are well established, with advantages including excipient safety, flexible formulation design, scalability in manufacturing, and the ability to reduce first pass metabolism. Their limitations include the risk of drug precipitation, oxidative instability of unsaturated lipids, and variability due to food effects. Despite these drawbacks, lipid systems have successfully reached the market, with examples such as Neoral^®^ (cyclosporine). Self microemulsifying drug delivery systems (SMEDDS) and their solid counterparts (S SMEDDS) provide fine droplet formation that enhances absorption, improved stability and handling through solidification, and scalability with optimized excipient ratios. Limitations include tolerability issues due to high surfactant content, risk of in vivo precipitation, oxidative instability, and the need for precise manufacturing control. Nevertheless, several marketed products validate this approach, including Norvir ^®^ (ritonavir) and Fortovase ^®^ (saquinavir) [65,66].

Pharmaceutical co crystals are characterized by precise solid state control, improved solubility without requiring high excipient loads, and compatibility with conventional tableting processes. Their limitations lie in the empirical nature of co former selection, risks of polymorphism, and challenges in maintaining supersaturation without precipitation. Despite these issues, co crystals have achieved regulatory success, with FDA approved products such as Entresto ^®^ (sacubitril/valsartan) [67,68].

Compared to lipid formulations, SMEDDS, and co crystals that already have marketed products, CA-based THEDES remain limited to research, with challenges in stability, safety, and manufacturability preventing their translation into clinical use. With caution, it should be noted that the enhanced solubilization folds reported for risperidone and gefitinib in CA THEDES systems have not been documented in the literature using other formulation strategies. However, the literature remains deficient in straightforward, systematic experimental comparisons, which are still needed to critically establish whether CA THEDES truly outperform established non eutectic approaches.

## 9. Safety, Toxicity, and Regulatory Considerations

The safety and biocompatibility of pharmaceutical excipients—both alone and in combination with APIs are critical determinants for whether a formulation can progress from the laboratory into clinical development and regulatory approval [69]. The safety of pharmaceutical excipients is highly dependent on the intended route of administration. Decanoic acid (CA) is recognized by the U.S. FDA as Generally Recognized As Safe (GRAS) when used in food product manufacturing or as a food additive under 21 CFR §§ 172.210, 172.860, 173.340, and 178.1010 [70].

When the API is combined with CA to form a DES, the molecular interactions between the components may modify their physicochemical and biological behavior. Consequently, each DES formulation is expected to exhibit a distinct safety profile that requires independent evaluation. However, the overall safety and toxicity of DESs have not yet been fully characterized, and the potential formation of degradation products from the API–CA system represents an additional concern that must be addressed [71].

In addition to acute safety considerations, it is important to distinguish between short-term tolerability and potential risks associated with repeated or long-term exposure to CA-THEDES formulations. While existing studies suggest favorable acute dermal compatibility, chronic exposure may pose different toxicological concerns, particularly given the intrinsic biological activity of medium-chain fatty acids such as CA. Regulatory agencies increasingly expect excipients with functional or bioactive roles to be evaluated beyond conventional inert excipient frameworks, including assessment of cumulative toxicity, local tolerability, metabolic fate, and potential systemic effects following prolonged use. At present, no standardized toxicological framework exists specifically for THEDES, underscoring the need for harmonized evaluation strategies encompassing long-term safety, degradation behavior, and exposure-dependent risk. Establishing such frameworks will be essential to support regulatory acceptance and facilitate the clinical translation of THEDES-based drug products. Previous evaluations of the risperidone–CA DES demonstrated favorable dermal compatibility, with no observable irritation or tissue damage. Histological analysis further confirmed the absence of inflammatory or structural alterations in the treated skin, supporting the formulation’s safety for topical application [27].

There is currently no dedicated regulatory guidance specifically for THEDES or products based on them. In the absence of such guidance, regulatory assessment relies on the nature of the API and the co-former. For CA-THEDES where the API is already approved and CA is a generally recognized as safe (GRAS) excipient, the system would likely be treated as an excipient-assisted formulation rather than a new chemical entity. In this scenario, the regulatory focus would be on Chemistry, Manufacturing, and Controls (CMC), bridging pharmacokinetic studies, and a full characterization of the finished dosage form, including composition, physical and chemical properties, stability, and validated analytical methods. Special attention may be needed for the container-closure system, as the eutectic liquid has the potential to extract materials, and impurities arising from the API or excipient must be addressed.

If the interaction between CA and the API significantly alters pharmacology, systemic exposure, or safety, the THEDES could be considered a new chemical entity, requiring a full Investigational New Drug (IND) or Investigational Medicinal Product Dossier (IMPD) package. This would include detailed nonclinical studies (pharmacology and toxicology), comprehensive CMC documentation, and clinical studies to support first-in-human use.

Unlike co-crystals and fixed-dose combinations, CA-THEDES are not simply regarded as solid-state modifications or conventional combinations of approved components. Co-crystals are generally recognized by the USFDA and EMA as distinct crystalline forms that do not introduce new pharmacological activity, while fixed-dose combinations are evaluated based on the established safety and efficacy of their individual active ingredients [72,73].

In contrast, CA-THEDES may exhibit altered physicochemical and biopharmaceutical behavior due to strong intermolecular interactions between the API and CA, which can influence drug performance. As a result, their regulatory assessment is expected to rely on a case-by-case evaluation, with emphasis on comprehensive characterization, pharmacokinetic bridging, and demonstration of safety and performance, rather than automatic classification as a new chemical entity.

## 10. Challenges and Future Perspectives

CA-THEDES face several barriers to pharmaceutical translation. Stability remains a key concern due to hygroscopicity, where moisture uptake can disrupt hydrogen-bonding networks and compromise physicochemical properties, drug solubility, and shelf-life. Although hydrophobic THEDES generally show improved stability compared with hydrophilic DESs, their long-term behavior under fluctuating humidity conditions remains insufficiently characterized [4,41]. In addition, despite reported thermal stability up to ~200 °C, oxidative degradation and compositional drift during storage necessitate systematic shelf-life evaluation [74].

High viscosity represents a major formulation and scale-up limitation. CA-THEDES typically exhibit higher viscosities than conventional solvents, restricting mass transfer and processability [27]. While viscosity can be partially reduced through temperature control or limited co-solvent addition, such strategies may perturb eutectic structure or drug solubility, requiring careful optimization [44,75,76]. These rheological constraints complicate industrial translation, where large-scale production demands strict control of mixing, temperature, and component quality, as well as pilot-scale validation of viscosity-dependent operations [8,77].

Substantial gaps also persist in toxicological, pharmacokinetic, and regulatory understanding. Although many individual components are classified as GRAS, their eutectic combination can produce emergent interactions that alter biological behavior, with growing evidence of formulation-specific cytotoxicity or biocompatibility profiles [41,78].

Regulatory progress is further impeded by the absence of clear classification frameworks distinguishing THEDES from related systems, alongside a lack of standardized assays for safety and biodegradability, limiting cross-study comparability and regulatory evaluation [79,80].

Future development is increasingly focused on multifunctional THEDES capable of synergistic drug co-delivery. Extending the principle of the lidocaine–prilocaine eutectic, CA-based systems have demonstrated enhanced therapeutic performance, exemplified by levofloxacin–CA-THEDES with improved antibacterial efficacy driven by combined solubility enhancement and intrinsic membrane-disruptive activity [40]. Artificial intelligence and machine learning are emerging as enabling tools to rationalize THEDES design by addressing formulation bottlenecks related to solubility, viscosity, and eutectic composition. ML models coupled with COSMO-RS descriptors enable accurate solubility prediction, while regression and neural-network approaches allow high-fidelity prediction of viscosity and melting behavior, reducing experimental screening burden [81,82,83,84,85,86].

Inverse-design and generative AI frameworks further support prioritization of optimized eutectic compositions, narrowing experimental design space rather than replacing empirical validation [87]. For AI-assisted THEDES development to achieve pharmaceutical relevance, models must remain interpretable and transparent, particularly in regulatory contexts, where explainable AI approaches such as SHAP analysis are increasingly employed to rationalize structure–property relationships and enhance confidence in AI-driven decisions [88,89,90,91]. In parallel, model performance depends critically on training with chemically representative and high-quality datasets [89,90,91], as over-representation of specific DES classes (e.g., choline chloride–based systems) can bias predictive accuracy [88,92,93], particularly against fatty-acid-based formulations such as CA-THEDES, thereby necessitating targeted dataset expansion to improve model generalizability and regulatory credibility [94,95]. Finally, because THEDES properties are sensitive to compositional ratios, water content, and raw-material variability, reproducible experimental workflows remain essential to ensure reliable AI training and cross-laboratory validation [96,97,98]. Addressing these formulation, regulatory, and data-driven challenges in parallel will be critical to advancing CA-THEDES toward clinical viability.

## 11. Conclusions

CA-based THEDES constitute a distinct and increasingly investigated class of biofunctional eutectic systems with applications extending beyond conventional solvent substitution. This review highlights that CA contributes a combination of directional hydrogen bonding, amphiphilic membrane interaction, and intrinsic biological activity, allowing it to function as a solubilizing agent, permeability modifier, and bioactive co-former within eutectic formulations. Across multiple studies, this multifunctional role is associated with measurable formulation-level improvements, including substantial solubility enhancement for poorly water-soluble drugs, synergistic antimicrobial or anticancer activity in selected systems, and improved transdermal or transmucosal transport relative to comparator formulations, achieved without reliance on volatile organic solvents.

Importantly, the influence of CA is structurally specific rather than generic. Its carboxyl group plays a central role in eutectic formation and drug amorphization, while the C10 aliphatic chain contributes to membrane interactions and microbial perturbation. Together, these features support the use of CA-THEDES as rationally designed pharmaceutical matrices capable of modulating drug solubility, stability, and transport, and of enabling alternative administration routes such as oral, transmucosal, or transdermal delivery.

Despite these advances, significant challenges remain, including the need for improved long-term stability assessment, viscosity management, comprehensive toxicological evaluation, and clearer regulatory classification. Emerging artificial intelligence- and machine learning-guided formulation strategies offer promising tools to address these issues by supporting rational eutectic partner selection, compositional optimization, and property prediction with greater efficiency and mechanistic transparency.

Overall, the available evidence positions CA-THEDES as a versatile platform within green pharmaceutical development, in which the solvent system contributes actively to formulation performance. Continued systematic investigation and standardization will be essential to define their translational potential and to clarify their role in the formulation of poorly soluble or permeation-limited therapeutic agents.

## Figures and Tables

**Table 1:** Comparative overview of eutectic systems (ES), deep eutectic solvents (DES), ionic liquids (ILs), and in situ deep eutectic systems (DESy).

Feature	ES	DES	ILs	DESy
Composition	Organic/inorganic blends of solid compounds	Mixtures of H-bond donors and acceptors (ionic or non-ionic)	Pure salts: discrete organic cations and inorganic/organic anions	In situ mixtures formed with the active compound as part of the eutectic system
Melting Behavior	Sharp melting point at eutectic composition (solid or semisolid at RT)	Depressed melting point; liquid at or near room temperature	Liquid at room temperature due to bulky asymmetric ions	Formed dynamically during application; liquid under process conditions
Type of Interactions	Weak van der Waals and minimal hydrogen bonding	Extensive hydrogen bonding network	Electrostatic (ionic) interactions	Hydrogen bonding and solvation driven by target molecule–solvent synergy
Polarity and Solubility	Moderate; limited to polar solutes	Polar; excellent for solubilizing poorly soluble APIs	Broad—dissolves both polar and non-polar compounds	Typically polar; designed to improve solubility of target APIs
Tunable Properties	Limited	Highly tunable via component selection and ratio adjustments	Highly tunable via cation/anion selection	Moderately tunable via selection of in situ interacting components and formulation conditions (e.g., temperature, water content).
Therapeutic Functionality	Typically absent	Possible if one or more components have inherent biological activity (THEDES)	Generally inert in drug delivery unless functionalized	Yes; active compound contributes to both therapeutic and solvent function
Toxicity and Biocompatibility	Depends on components	Often low (especially with natural components like fatty acids, amino acids, sugars)	Variable; some ILs have cytotoxicity and environmental concerns	Generally favorable if composed of GRAS or natural components
Environmental Impact	Moderate to high (based on solvents used)	Low (especially for NaDES and THEDES based on natural compounds)	Often high; requires careful design for biodegradability	Low; reduced processing steps and mild preparation conditions
Example Systems	Benzoic acid–urea	Choline chloride–urea, choline chloride–CA, menthol–ibuprofen	1-butyl-3-methylimidazolium chloride ([Bmim] Cl), [EtPy] [BF_4_]	In situ systems formed with kiwifruit or date seed polysaccharides
Applications	Melting point depression, food, metallurgy	Green extraction, pharmaceutical formulation, topical/transdermal delivery (THEDES)	Organic synthesis, catalysis, electrochemistry, solubilization	Prebiotic extraction, drug solubilization, biocompatible formulations
Reference	[22]	[23]	[24]	[19]

**Table 2:** Representative CA-based eutectic and THEDES, summarizing eutectic partners, composition ranges, physicochemical behavior, and key functional outcomes across pharmaceutical and benchmark applications.

Components with CA	Ratio	Observation	Application	Reference
Tetradecanoic acid (myristic acid)	CA: Myristic acid = 82:18 (mol%) or 78:22 (wt%)	Smooth, homogeneous, congruent melt; no phase separation	As a phase change material (PCM) of potential interest for passive temperature control in buildings	[43]
Myristic acid, Lauric acid, Steric acid	Molar Ratio (CA: Myristic acid) 3:1	Pasty-like solid	Synergistic antimicrobial activity	[39]
	Molar Ratio (CA: Lauric acid) 2:1	Transparent liquid		
	Molar Ratio (CA: Stearic acid) 4:1	White solid		
Thymol, Menthol	(Thymol: CA) 0.33:0.67	Homogeneous liquid	Reported physical properties and their dependence on constituents/composition of the NADESs will enhance their utility and help establish them as novel alternate media in science and technology. (Menthol: CA)	[44]
	(Thymol: CA) 0.50:0.50	Homogeneous liquid		
	(Thymol: CA) 0.67:0.33	Homogeneous liquid		
	(Menthol: CA) 0.33:0.67	Homogeneous liquid		
	(Menthol: CA) 0.50:0.50	Homogeneous liquid		
	(Menthol: CA) 0.67:0.33	Homogeneous liquid		
Gefitinib	(Gefitinib: CA) 80:20	Clear liquid	Enhance Gefitinib solubility and exhibit a synergistic cytotoxic effect against EGFR-expressing cell lines	[6]
	(Gefitinib: CA) ≤70:30	Pasty		
	(Gefitinib: CA)Extreme ratios	Powder/solid		
Tetrabutylammonium chloride (TBAC), methyl tricaprylmethylammonium chloride (TOMAC)	(TBAC:CA) 1:2	Clear, viscous fluid	Have the potential to be a novel class of lubricants	[45]
	(TOMAC:CA) 1:2	Clear, viscous fluid		
Cineole	(Cineole: CA) 1: 1	Clear, low-viscosity liquid	Very low viscous and dense fluid, with suitable properties for several solubilization technologies. its suitability to penetrate and stabilize cell membranes may lead to adverse outcomes when living organisms are exposed to this hydrophobic deep eutectic solvent.	[26]
Droperidol	(Droperidol: CA) 0.9:0.1 (D1)	Clear eutectic liquid; ^1^H NMR showed Δδ = +0.08–0.09 ppm at protons adjacent to piperidine N; DOSY confirmed reduced CA diffusion; DSC showed no melting peaks; highest intestinal flux (1.182 mg cm^−2^ s^−1^ at 15 min).	Solvent-free THEDES platform for enhancing droperidol solubility and intestinal permeability; suitable for green pharmaceutical formulation.	[8]
	(Droperidol: CA) 0.8:0.2 (D2)	Homogeneous liquid; similar NMR shifts; DSC revealed CA recrystallization on cooling (−8.1 °C); slightly lower flux than D1.		
	(Droperidol: CA) 0.7:0.3 (D3)	Partial melting; depressed CA melting at 17.7 °C in DSC; signs of phase separation.		
	(Droperidol: CA) ≤ 0.6:0.4 (D4–D8)	Pasty mixtures; recrystallization and Tg observed in DSC; weak interaction.		
Aripiprazole	(Aripiprazole: CA) 0.9:0.1 (A1)	Clear eutectic liquid; downfield shifts in amide (Δδ = +0.62 ppm) and piperazine CH_2_ (Δδ = +0.10 ppm); strong H-bonding confirmed.	Hydrogen-bond-stabilized eutectic systems enabling improved solubility and biopharmaceutical performance of aripiprazole.	[8]
	(Aripiprazole: CA) 0.8:0.2 (A2)	Similar spectral shifts: eutectic liquid maintained; no residual crystallinity.		
	(Aripiprazole: CA) ≤ 0.7:0.3 (A3–A8)	Heterogeneous pastes; DSC showed unincorporated CA (e.g., CA melting at 18.7 °C in A3); weak or absent interactions.		
Lauric acid	(Lauric acid: CA) 1:2	Clear, homogeneous liquid; lowest density (0.859 g/cm^3^); Newtonian flow; visually stable. Lycopene yield 7.51 mg/100 g FW, total carotenoids 8.04 mg/100 g.	Green solvent for lycopene extraction; practical operating window established.	[46]
Lauric acid	(CA: Lauric Acid) 1:1	Transparent liquid; thicker flow (shear-thickening). Lycopene 2.98 mg/100 g.	Alternative HNADES with moderate performance.	[46]
	(CA: Lauric Acid) 2:1	Transparent liquid; more viscous feel; Lycopene 3.19 mg/100 g.	Lower-performing variant.	
Dodecanoic acid	(Dodecanoic cid:CA) 1:2	Clear, uniform liquid; noticeably viscous; stable hydrophobic phase. Effective for Cu^2+^, Co^2+^, Ni^2+^ extraction	Green solvent for metal recovery; applicable in wastewater treatment.	[47]
Matrine	(Matrine:CA) 1:1	Slightly yellowish homogeneous liquid; density and viscosity decrease with temperature; moderate thermal stability	better antibacterial activity on *S. aureus* as compared with matrine	[48]
Polyethylene glycol	Weigh% Mass Ratio (polyethylene glycol:CA) 1:1	Congruent eutectic at ~22.9 °C with high latent heat (173.9 J g^−1^); reduced supercooling, faster crystallization, stable after 200 cycles, negligible corrosion.	Thermal energy storage for solar passive buildings; energy saving (≈4.9 kWh·kg^−1^·yr^−^^1^), cost-effective, and carbon neutral within ~3 years.	[49]
Mirtazapine	(Mirtazapine:CA) 1:2	Light-yellow transparent viscous liquid; no crystals (polarized microscopy)	Transdermal delivery of MTZ to bypass first-pass metabolism; promising topical antidepressant THEDES.	[50]
Levofloxacine:	(CA:Levofloxacin) 9:1, 8:2, 7:3 (DES liquids formed at these ranges; eutectic ~80:20–70:30	Clear liquids (THDES); DES formation confirmed by ^1^H NMR and ATR-FTIR (H-bonding) and DSC (melting point depression; excess CA signal decreases with more LEV).	Green THDES to enhance LEV performance: solubilization + antibacterial synergy; potential to combat resistance.	[40]
Ketoconazole:	Molar Ratio (Ketoconazole: CA) 1:5	Clear, stable liquid at room temperature for ≥ 5 months.	Enhanced antifungal efficacy, solubility, and transdermal permeability using a green, stable THEDES system.	[7]
Oxymatrine:	(Oxymatrine: CA) 1: 1	Stable transparent DES.	Biocompatible, low-toxicity enhancer; suitable when high safety is prioritized.	[51]
Ibuprofen:	(Ibuprofen: CA) 1:3	Clear, stable liquid at 37 °C.	Effective transient eutectic solubilizer; excellent for dual-drug oral systems when combined with; balances high solubility and moderate release.	[52]
Clotrimazole	(Clotrimazole:CA) 1:1	Solid at RT (not a DES)	Therapeutic DES (THEDES) for enhanced antifungal potency and skin permeation	[53]
	(Clotrimazole:CA) 1:2	Transparent liquid; physically stable ≥ 1 year		
	(Clotrimazole:CA) 1:3	(DES candidate) Eutectic point; transparent liquid		
	(Clotrimazole:CA) 1:4	Transparent liquid; physically stable (DES candidate)		
	(Clotrimazole:CA)1:5	Solid at RT (not a DES)		

**Table 3:** Physicochemical Properties of Selected Saturated Fatty Acids.

Fatty Acid	Carbon No.	Melting Point (°C)	Relevant Viscosity	Aqueous Solubility (g/L) at 20 °C
Octanoic acid	C8	16.7	Low	0.7
Nonanoic acid	C9	12.5	Low	0.3
CA	C10	31.6	Low-moderate	0.15
Lauric acid	C12	44.2	Moderate–high	0.055
Myristic acid	C14	53.9	High	0.02
Palmitic acid	C16	63.1	High	0.007
Stearic acid	C18	69.6	Very high	0.003

## Data Availability

No new data were created or analyzed in this study.

## Abbreviations

The following abbreviations are used in this manuscript:

CA	Capric acid
ILs	Ionic Liquids
THEDES	Therapeutic Deep Eutectic Systems
DES	Dee Eutectic Solvent
DESy	Deep Eutectic System
